# The effects of inhibition of fatty acid amide hydrolase (FAAH) by JNJ-42165279 in social anxiety disorder: a double-blind, randomized, placebo-controlled proof-of-concept study

**DOI:** 10.1038/s41386-020-00888-1

**Published:** 2020-10-18

**Authors:** Mark E. Schmidt, Michael R. Liebowitz, Murray B. Stein, Jennifer Grunfeld, Ilse Van Hove, W. Kyle Simmons, Peter Van Der Ark, James A. Palmer, Ziad S. Saad, Darrel J. Pemberton, Luc Van Nueten, Wayne C. Drevets

**Affiliations:** 1 Janssen Research & Development, Beerse, Belgium; 2 The Medical Research Network, New York, NY USA; 3 University of California San Diego, La Jolla, CA USA; 4 Peninsula Therapeutic & Research Group, Frankston, VIC Australia; 5 Janssen Research & Development, La Jolla, CA USA; 6 Oklahoma State University Brain Imaging Center, Tulsa, OK USA

**Keywords:** Drug development, Anxiety

## Abstract

JNJ-42165279 is a selective inhibitor of fatty acid amide hydrolase (FAAH), the enzyme responsible for the degradation of fatty acid amides (FAA) including anandamide (AEA), palmitoylethanolamide (PEA), and N-oleoylethanolamide (OEA). We assessed the efficacy, safety, tolerability, pharmacokinetics, and pharmacodynamics of treatment with JNJ-42165279 in subjects with social anxiety disorder (SAD). This was a multicenter, double-blind, placebo-controlled study randomizing subjects to 12 weeks of treatment with either JNJ-42165279 (25 mg daily) or placebo (PBO). The primary endpoint was the change in the Liebowitz Social Anxiety Scale (LSAS) total score from baseline to end of study. Secondary endpoints included the Hamilton Anxiety Scale (HAM-A), Hamilton Depression Rating Scale (HDRS_17_), and the Clinical Global Impression-Improvement (CGI-I). Samples were collected for plasma concentration of AEA, PEA, OEA, and JNJ-42165279. A total of 149 subjects were enrolled with a mean baseline LSAS total score of 102.6 (SD 16.84). The mean change from baseline (SD) in LSAS total score at week 12 was numerically greater for JNJ-42165279: −29.4 (27.47) compared to PBO: −22.4 (23.57) but not significant. The percentage of subjects with ≥30% improvement from baseline in the LSAS total score was significantly higher for JNJ-42165279 (42.4%) compared to PBO (23.6%) (*p* value = 0.04). The percentage of subjects with a CGI-I score of much or very much improved was also significantly higher for JNJ-42165279 (44.1%) than for PBO (23.6%) (*p* value = 0.02). The drug was well tolerated. JNJ-42165279 appears to elicit an anxiolytic effect in subjects with SAD although trough concentrations with 25 mg once daily appeared to be insufficient to completely inhibit FAAH activity which may have led to suboptimal efficacy. ClinicalTrials.gov Identifier: NCT02432703.

## Introduction

The endocannabinoid system is thought to participate in the regulation of fear and anxiety responses, the immune system, and pain perception. N-arachidonoylethanolamine (anandamide) was one of the earliest identified endocannabinoids and acts as low-efficacy agonist at CB1 receptors [1, 2]. Unlike classical neurotransmitters synthesized and stored in neurons until release, anandamide is produced on demand from selected membrane phospholipids and released from cells. In the nervous system, anandamide is produced following postsynaptic activation and can act as a retrograde inhibitor of neuronal activity via its binding to presynaptic CB1 receptors [3]. After reuptake by cells, anandamide and other fatty acid amides (FAA) are rapidly inactivated by enzymatic hydrolysis. The principal clearance enzyme for anandamide is fatty acid amide hydrolase (FAAH), which is expressed in a number of tissues and highly expressed in the brain. JNJ-42165279 is a potent, selective, and orally bioavailable inhibitor of FAAH [4]. The compound is a substrate of the enzyme and inhibits its activity by covalent binding to the catalytic site. Enzyme activity is restored via slow hydrolysis of the covalently bound drug fragment from the active site and regeneration of enzymatically active FAAH. The dose related peripheral and central pharmacokinetic (PK) and pharmacodynamic (PD) properties, safety, and tolerability of JNJ-42165279 have been evaluated in multiple Phase 1 studies [5].

Social anxiety disorder (SAD) is a common anxiety disorder and is associated with significant distress and dysfunction in affected individuals [6, 7]. The disorder can persist throughout life and is characterized by exaggerated fear of being negatively evaluated in social situations and heightened anxiety during social interactions [8]. SAD is a risk factor for major depressive disorder (MDD) and anxiety disorders such as generalized anxiety disorder (GAD) are frequently comorbid [9]. Symptoms can be ameliorated, and function improved by a number of treatments including selective serotonin reuptake inhibitors (SSRIs) and other antidepressants, benzodiazepines, and pregabalin as well as cognitive and behavioral therapies [10]. Each of these has limitations, most notably poor tolerance to sexual adverse effects associated with SSRIs and other antidepressants, and sedation/risk for tolerance and abuse with benzodiazepines. Moreover, a considerable number of subjects with SAD respond poorly to available treatments and remain highly symptomatic [11]. Thus, there continues to be a need for effective, safe, and well tolerated pharmacological treatments.

Modulation of anandamide concentrations by inhibiting FAAH can reduce anxiety behaviors in rodent models of chronic stress [12]; specifically enhances fear extinction in rodent models [13]; facilitates fear extinction and attenuates autonomic and subjective stress responses to the Maastricht Acute Stress test in healthy volunteers [14]. To date, the anxiolytic effects of FAAH inhibitors in clinical populations have not been reported. We elected to test whether treatment with JNJ-42165279 could reduce symptom burden in subjects with SAD as reflected by change in the Liebowitz Social Anxiety Scale (LSAS) total score from baseline to end of study as the primary endpoint. Moreover, the occurrence of comorbid MDD and GAD in subjects with SAD could allow for exploration of broader effects in mood and anxiety disorders.

## Methods

This was a multicenter, double-blind, placebo (PBO)-controlled, randomized, parallel-group Phase 2a proof-of-concept study to assess the efficacy, safety, and tolerability of JNJ-42165279 over 12 weeks of treatment. The aim of the study was to determine whether an anxiolytic effect could be identified as a result of FAAH inhibition with JNJ-42165279 in subjects with SAD. Participants were enrolled and randomly assigned in a 1:1 ratio to either 25 mg of JNJ-42165279 or matching PBO capsules. The plasma half-life of JNJ-42165279 is 8–14 h. As the compound is a slowly reversible inhibitor, FAAH inhibition persists following clearance of the parent drug supporting once daily dosing. The 25 mg dose was predicted to inhibit FAAH enzyme in the brain throughout the dosing interval, based on the outcome of the Phase 1 studies [5]. Investigators from the USA (11 sites), Australia (5 sites), and Canada (4 sites) participated in the trial. The trial was initiated in June 2015 and completed in August 2018. The trial was placed on temporary hold from January 2016 until December 2016 after a healthy volunteer enrolled in a Phase 1 study of BIA 10-2474 (Bial Pharmaceutical company, Trofa, Portugal) unfortunately passed away following a hemorrhagic stroke. Subjects in the same cohort were also hospitalized with described possible brain tissue damage [15]. BIA 10-2474 was reported to be an FAAH inhibitor and the FDA placed a hold on all clinical studies with FAAH inhibitors pending investigation. Following a detailed investigation of the Bial compound and the clinical study, it was determined that the adverse events (AEs) were not related to FAAH inhibition [16] and the clinical hold on FAAH inhibitors was subsequently lifted.

Male and female subjects with a primary DSM-5 diagnosis of SAD, who were between 18 and 64 years of age (inclusive) were eligible for enrollment, provided that they displayed a minimum symptom severity as measured by the LSAS score of ≥70 at screening and for whom pharmacotherapy was indicated. Initially the only female subjects able to be enrolled were women who were unable to bear children as an acceptable safety margin at this stage had not been identified. Once these data were available, the protocol was amended to permit enrollment of childbearing potential women, provided they were using a highly effective method birth control method. Subjects with comorbid GAD or MDD were able to participate provided SAD was considered the primary diagnosis and the subject had a current Hamilton Depression Rating Scale (HDRS_17_) total score ≤18 at screening.

Subjects with the DSM-5 performance-only specifier for SAD or with other ongoing significant psychiatric conditions, including, but not limited to MDD with psychotic features, bipolar disorder, obsessive-compulsive disorder, borderline personality disorder, eating disorder, autism spectrum disorders, posttraumatic stress disorder, or schizophrenia were excluded. Subjects with a history of drug or alcohol use disorder within 6 months prior to screening, positive test result(s) for drugs of abuse, or significant medical illnesses were excluded. This was a monotherapy trial; no antidepressant or anxiolytic medications were allowed and subjects who had failed more than two adequate pharmacological treatment trials for SAD, defined as lack of response to at least 10 weeks of treatment at adequate doses were excluded. Participants could not be participating in evidence-based psychotherapy for SAD although other forms of psychotherapy not focused on SAD (for example 12 step programs) were permitted. Standardized behavioral instructions were provided by the investigator. At the beginning of the trial, subjects were advised to enter socially feared situations to help determine whether the treatment has beneficial or adverse effects on symptoms and behaviors and at subsequent visits the investigator asked subject about social situations that he/she had encountered since the last visit, and how he/she felt in those situations.

The study consisted of three phases: a 28-day screening phase, a 12-week double-blind treatment phase, and a follow-up visit between 7 and 28 days following the last dose of drug treatment. All subjects who provided a written informed consent and were considered eligible for the study entered the double-blind treatment phase on Day 1. Visits were scheduled at 1, 2, 4, 6, 8, 10, and 12 weeks after enrollment. During the entire blinded treatment period, subjects self-administered once daily the assigned study agent (JNJ-42165279 or PBO) in the morning following breakfast.

During the treatment phase, safety, and tolerability were monitored at regular intervals (AEs reporting, physical examination, suicidality risk assessment, vital signs, 12-lead electrocardiogram, and clinical labs including drug and alcohol screens and urine pregnancy tests). Blood samples were collected at specified time points to evaluate the PK (days 14, 28) and PD effects (days 1, 28, 84: plasma FAA concentrations) of JNJ-42165279. A pharmacogenomic blood sample was collected from all eligible subjects to determine whether the subject had one or more copies of the FAAH gene rs324420 385C allele [17]. The methods for biomarker sample collection and processing and assays are provided in the Supplementary material.

The protocol was approved by the institutional review boards for each of the clinical sites and all subjects provided written informed consent. The protocol was amended following the clinical hold to include periodic neurological examinations to confirm the compound safety.

### Measures

The Mini International Neuropsychiatric Interview (English Version 7.0.0 for the DSM-5) was administered by a trained rater to determine the primary diagnosis and presence/absence of any co-existing psychiatric conditions. The LSAS was used to determine the symptom severity and as the primary treatment endpoint. The LSAS scale consists of 24 items which are divided into 2 subscales that address social interaction (11 items) and performance (13 items) situations. An overall total score was calculated by summing the 24 fear and 24 avoidance scores with a maximum score of 144, with higher scores indicating higher severity of SAD [18]. The HDRS_17_ [19] and Hamilton Anxiety Scale (HAM-A) [20] were used to assess severity of depression and anxiety during the trial and as secondary endpoints, both were administered using structured interview guides. Rater training on the LSAS was conducted by MRL and screening interviews were centrally reviewed for adherence with protocol inclusion and exclusion criteria.

The primary efficacy endpoint was improvement in social anxiety symptoms, as measured by change in the LSAS total score from baseline to the 12-week endpoint. The secondary endpoints included changes from baseline to the 12-week endpoint for: LSAS fear/anxiety subscale, LSAS avoidance subscale, LSAS ≥ 30% and ≥50% improvement from baseline on total score, HAM-A total score, HAM-A ≥ 50% improvement from baseline on total score, HDRS_17_ total score, HDRS_17_ anxiety/somatization factor score, and the Clinical Global Impression-Improvement (CGI-I) score [21].

Exploratory endpoints included changes in Sheehan Disability Scale [22], GAD-7 [23], Snaith–Hamilton Pleasure Scale [24], Medical Outcomes Study Sleep-revised (MOS Sleep R) [25], Quality of Life Enjoyment and Satisfaction Questionnaire [26], and a Self-Assessment of Treatment Experience.

### Statistical methods

The sample size for the study assumed a treatment difference of at least 10 points in the mean change from baseline to the endpoint in LSAS total score between JNJ-42165279 treatment group and PBO. A standard deviation (SD) of 24 in the change in LSAS total score from baseline was used based on published data [11, 27, 28]. To detect a treatment difference of 10 points with a power of 90% at an overall 1-sided significance level of 0.20, 53 subjects in each group were required. An alpha of 0.20 was chosen to balance between a type 1 error (false positive) and type 2 error (false negative) by increasing sensitivity for detecting a therapeutic signal while also maintaining a reasonable and modest sample size. Thus, power was set to a high value (power = 90%; beta = 10%), but the type 1 error rate was specified at 1-sided alpha = 0.20 and used to reduce the risk of rejecting a compound with therapeutic potential in an early stage of development [29]. When adjusted for a drop-out rate of ~15% of subjects, the required number of subjects was 61 per treatment group. The total number of subjects entering the study was increased by 15 subjects to replace those who were prematurely stopped when the study was temporarily suspended.

All efficacy analyses were based on the intention-to-treat (ITT) analysis set defined as all randomized subjects who received at least one dose of study agent (either PBO or JNJ-42165279) and had at least one assessment in the double-blind treatment phase on any of the efficacy parameters. Subjects who withdrew early from the study at the time of study suspension were not included in the ITT analysis set (seven subjects in the PBO and eight subjects in the JNJ-42165279 groups). Subjects who had already completed the study at the time it was suspended, however, were included in the ITT set (nine subjects in the PBO and nine subjects in the JNJ-42165279 groups).

The JNJ-42165279 treatment group was compared with the PBO group for the primary efficacy endpoint (change from baseline in total LSAS score during the double-blind treatment phase) by means of a mixed-effects model using repeated measures (MMRM), with time, treatment, and time-by-treatment interaction as factors, baseline total LSAS score and age as a continuous covariate, and country, and the presence of comorbid MDD as categorical covariates. An unstructured variance covariance matrix was used. The treatment-PBO differences were obtained using the appropriate contrast in the MMRM models at the 12-week endpoint. The change from baseline for the secondary continuous efficacy endpoints were analyzed in the same manner as for the LSAS total score. Exploratory endpoints were analyzed using descriptive statistics only. Sensitivity analyses of the primary efficacy endpoint were performed using an analysis of covariance model. Descriptive statistics for values and changes from baseline (where applicable) were provided by treatment group for all efficacy measures, including subscale scores for selected scales, at each time point of the double-blind treatment phase. Frequency of ≥30% and ≥50% improvement of social anxiety symptoms (derived from LSAS), as well as response frequency of depressive and anxiety symptoms (derived from the HDRS_17_ and HAM-A) and CGI-I were determined by treatment group at each time point of the double-blind treatment phase. Chi-square test was used to test the overall difference between the treatment groups in ≥30% and ≥50% improvement in LSAS total score and CGI-I response at week 12.

## Results

**Fig. 1:**
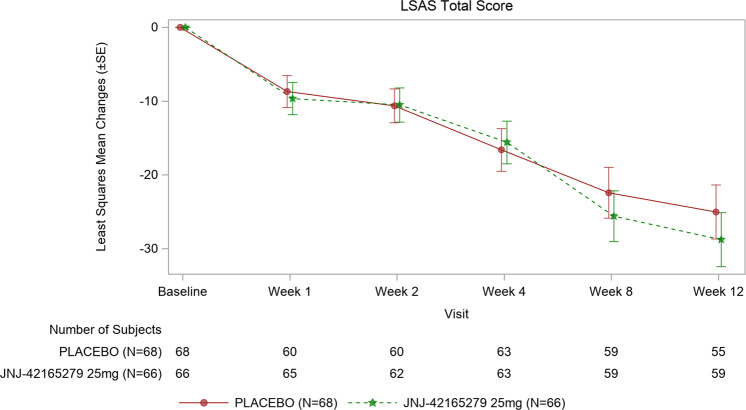
Least squares mean changes (±SE) from baseline of the LSAS total. Change in the LS means of LSAS total scores from baseline over time to week 12; ITT analysis set.

At the completion of enrollment, a total of 149 subjects (JNJ-42165279 *N* = 74, PBO *N* = 75) were included in the safety analysis. The mean ± SD age was 37.8 ± 13.07 years; 65% were male. In total, 10% had comorbid MDD and 16% had comorbid GAD. The mean ± SD baseline LSAS total score for JNJ-42165279 was 100.4 ± 16.79 and for PBO was 104.7 ± 16.72 (severe) (Table 1). Subjects who withdrew prematurely from the study at the time of study suspension were not included in the ITT analysis set for efficacy (seven subjects in the PBO group and eight subjects in the JNJ-42165279 group). Subjects who had already completed the study at the time it was suspended, however, were included in the ITT set (nine subjects in each group). A total of 134 subjects were included in the ITT set (JNJ-42165279 *N* = 66, PBO *N* = 68) (Fig. 1S, CONSORT diagram Supplementary online material (SOM)).

**Table 1:** Demographics.

	PBO	JNJ-42165279	Total
Number	75	74	149
Age (years) mean (SD)	37.4 (13.65)	38.3 (12.53)	37.8 (13.07)
Gender			
Male	49 (65.3%)	48 (64.9%)	97 (65.1%)
Female	26 (34.7%)	26 (35.1%)	52 (34.9%)
Race			
White	45 (60.0%)	50 (67.6%)	95 (63.8%)
Black/African American	15 (20.0%)	18 (24.3%)	33 (22.1%)
Asian	8 (10.7%)	4 (5.4%)	12 (8.1%)
Country			
AUS	11 (14.7%)	12 (16.2%)	23 (15.4%)
CAN	14 (18.7%)	12 (16.2%)	26 (17.4%)
USA	50 (66.7%)	50 (67.6%)	100 (67.1%)
LSAS mean (SD)	104.7 (16.72)	100.4 (16.79)	102 (16.84)
HAM-A mean (SD)	10.6 (7.47)	10.0 (7.34)	10.3 (7.39)
HDRS17 mean (SD)	6.7 (4.63)	6.4 (4.88)	6.5 (4.74)
Comorbid MDD	8 (10.7%)	7 (9.5%)	15 (10.1%)
Comorbid GAD	11 (14.7%)	13 (17.6%)	24 (16.1%)

The LSAS total score decreased steadily over time, with improvements in both JNJ-42165279 and PBO treatment groups. The mean change from baseline ±SD in LSAS total score at week 12 was −29.4 ± 27.47 for subjects in JNJ-42165279 group and −22.4 ± 23.57 for subjects in PBO group. Based on an MMRM model with time, treatment, country, presence of comorbid MDD, and time-by-treatment interaction as factors, baseline LSAS total score and age as a continuous covariate, and a random subject effect, the LS mean difference ±standard error (SE) between JNJ-42165279 and PBO groups was −3.8 ± 4.72. The difference between treatment groups was not statistically significant (1-sided *p* = 0.213 with 60% confidence interval [CI]: −7.76 to 0.22, Cohen’s *D* = 0.15) (Fig. 1). The similar results were also observed for the LSAS fear/anxiety and avoidance subscale scores (fear/anxiety subscale LS mean difference ±SE between JNJ-42165279 and PBO: −2.2 ± 2.40, *p* = 0.19; Avoidance subscale LS mean difference ±SE between JNJ-42165279 and PBO: −1.8 ± 2.44, *p*= 0.23). An analysis that censored subjects with no detectable drug concentrations (11 out of 59 in the JNJ-42165279 group) revealed a larger change from baseline (−30.2 ± 28.2) for JNJ-42165279 and −22.4 ± 23.6 for PBO; least-square mean difference ±SE (−4.5 ± 5.03), which met the protocol statistical threshold for effect (*p* = 0.19, one-sided) but was small (Cohen’s *D* = 0.18).

### Secondary endpoints

**Fig. 2:**
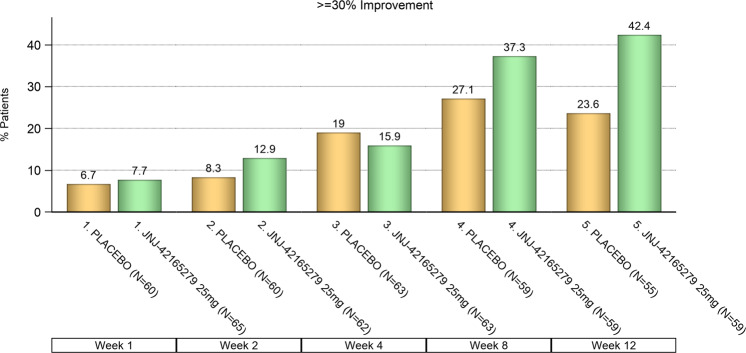
LSAS ≥ 30% improvement from baseline on total score: frequency distribution over time. The percentage of subjects who have ≥30% improvement from baseline in the LSAS total score was higher for JNJ-42165279 than for PBO, *p* value of 0.0348 (odds ratio: 2.4; 95% CI: 1.06–5.33, Chi-square); ITT analysis set.

The percentage of subjects who had a ≥30% improvement from baseline in the LSAS total score following 12 weeks of treatment was significantly higher for JNJ-42165279 (42.4%) compared to PBO (23.6%) (*p*= 0.03; Fig. 2). The percentage of subjects who had ≥50% improvement from baseline in LSAS total score was higher for JNJ-42165279 (18.6%) than for PBO (12.7%), although the difference was not statistically significant (*p* = 0.39).

**Fig. 3:**
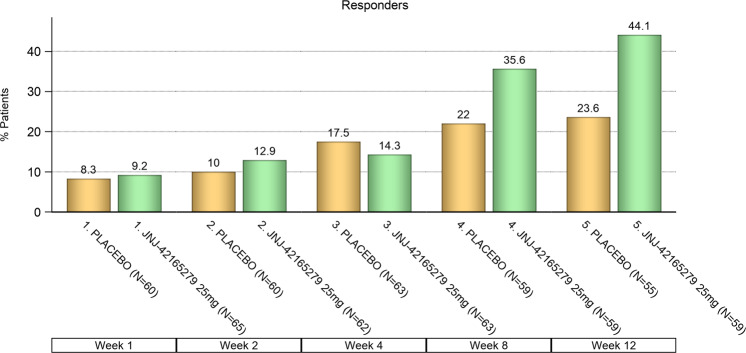
CGI-I: frequency distribution of responders over time. The percentage of subjects with a CGI-I score of “very much improved” or “much improved” was higher for JNJ-42165279 than for PBO, *p* value = 0.02 (odds ratio: 2.5, 95% CI: 1.14–5.70, Chi-square); ITT analysis set.

CGI-I: the percentage of subjects with a CGI-I score of “very much improved” or “much improved” was statistically significantly higher at the end of 12 weeks for JNJ-42165279 (44.1%) than for PBO (23.6%) (*p* = 0.02; Fig. 3). Improvement on the CGI-I was highly concordant with ≥30% improvement from baseline in the LSAS total score *p* < 0.0001, (Table 2S, SOM). The results from other secondary and exploratory endpoints are summarized in Tables 3S, 4S, and 5S, SOM.

Sixteen percent of the sample met diagnostic criteria for GAD. A larger treatment effect on the LSAS total score was seen in subjects with comorbid GAD (−35.0 ± 7.26 for JNJ-42165279 and −18.4 ± 6.96 for PBO, LS mean difference −16.6 ± 10.09, *p* = 0.059, Cohen’s *D* = 0.70) (Table 4S SOM). A significant treatment effect was seen on the GAD-7 (LS mean difference −1.9 ± 0.82, *p* = 0.01, Table 5S SOM). There was a modest reduction in anxiety symptoms on the HAM-A which met the statistical threshold for effect (*p* = 0.19) but was not clinically meaningful. Ten percent of the sample had comorbid MDD, and depression severity on the Hamilton depression scale was low at baseline. Little change in symptom severity occurred over the trial and there was no significant treatment effect although there was not much room to demonstrate a change (Table 3S, SOM).

### Biomarkers

**Fig. 4:**
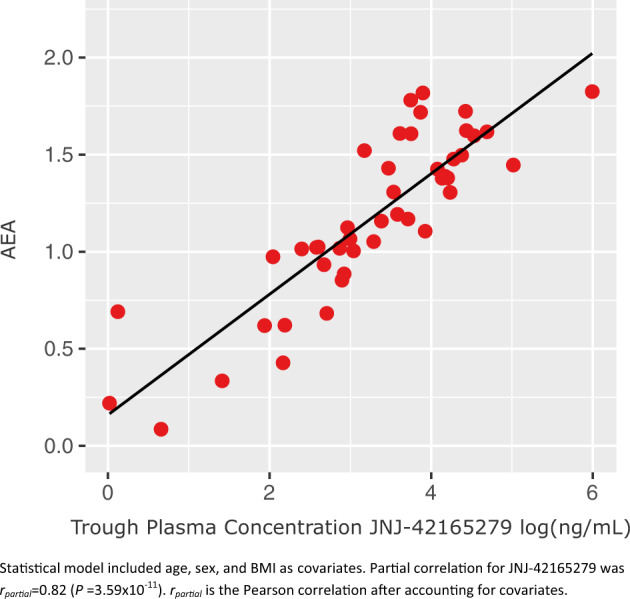
Relationship between plasma anandamide (AEA) and trough concentrations of JNJ-42165279 at 4 weeks. The statistical model included age, sex, and BMI as covariates. Partial correlation for JNJ-42165279 was *r*_partia*l*_ = 0.82 (*p* = 3.59 × 10^−11^). *r*_partial_ is the Pearson correlation after accounting for covariates.

Treatment with JNJ-42165279 was associated with increases in plasma anandamide (AEA), palmitoylethanolamide (PEA), and N-oleoylethanolamide (OEA) concentrations as anticipated (Fig. 2S SOM). Trough plasma concentrations of JNJ-42165279 and AEA at 4 weeks were strongly correlated (*r*_partial_ = 0.82, *p* = 3.59 × 10^−11^) (Fig. 4). Similarly, week 4 OEA and PEA levels were strongly positively correlated with trough plasma concentrations of JNJ-42165279 with *r*_partial_ of 0.85 (*p* = 3.59 × 10^−11^) and 0.77 (*p* = 1.24 × 10^−7^), respectively. The relationship between plasma concentrations of AEA and change from baseline in the LSAS at 12 weeks was explored post hoc by separating the JNJ-42165279 sample into tertiles of AEA concentrations at 12 weeks. Treated subjects with the most elevated AEA (3rd tertile) at week 12 exhibited the numerically greatest mean change from baseline with a 12.9 point greater change compared to PBO (Fig. 3S SOM).

### Genetics

Samples were tested for the rs324420 genotype in 141 subjects. The A/A genotype was present in 11 subjects (8%); A/C in 41 (29%); and C/C in 89 (63%). Baseline FAAs (AEA, PEA, OEA) were significantly higher in the A/A group compared to the other 2 groups. The results of the genetic analysis of plasma FAA concentrations at baseline are in Table 6S, SOM.

### Safety

The drug was well tolerated, and no notable neurological AEs of interest or findings occurred in either treatment groups (Table 5S SOM for AEs reported). No deaths occurred, and most AEs were mild to moderate in severity. Insomnia was more common among subjects on PBO and sleep quality was reported as significantly better by subjects on JNJ-42165279 on the MOS Sleep R (nine item) (Table 3S, SOM). Two serious AEs occurred: a subject admitted for alcohol use disorder and a subject who experienced an anaphylactic reaction due to accidental exposure to a food allergen, neither was attributed to study drug. Mean changes from baseline in hematology, serum chemistry, and urinalysis parameters were minimal, and were similarly distributed in the PBO and JNJ-42165279 treatment groups, with none considered clinically relevant. Transient elevations in ALT occurred in two subjects, both assigned to PBO.

## Discussion

Inhibition of FAAH has long been proposed to have potential as a treatment for anxiety disorders based on effects in preclinical models [12] but evidence of an effect in clinical disorders has not been reported to date. FAAH inhibition and the resulting elevation of AEA has been reported to facilitate fear extinction in rodents and humans [13, 14]. Fear is a major component of SAD such as fear of being singled out, rejected, or humiliated in social settings leading to avoidance and withdrawal. On the other hand, benzodiazepines are reported to interfere with retrieval of fear learning [30] and chronic SSRI treatment has been reported to impair fear extinction [31] in preclinical models, while both medication classes are effective in reducing symptom severity in SAD. Thus, the effects we observed may not depend on increased AEA enhancement of fear extinction and could be related to other mechanisms. This trial was conducted as a proof-of-concept study to determine whether a therapeutic effect could be detected in SAD that would support further development of JNJ-42165279 and exploration in other anxiety and stress disorders.

Treatment of SAD with JNJ-42165279 for 12 weeks did not result in a significant difference in mean change from baseline symptom severity on the LSAS compared to PBO, the primary endpoint for this study. There was also no effect on functional impairment as assessed by the Sheehan Disability Scale for which significant effect sizes are typically seen with these treatments [28, 32, 33]. Notably, however, twice as many subjects on JNJ-42165279 experienced at least a 30% improvement from baseline on the LSAS and twice as many subjects treated with JNJ-42165279 were assessed clinically as having improved on the CGI-I, similar to the proportion observed in a trial with sertraline [33].

Given the effects of JNJ-42165279 seen on the CGI-I compared to PBO, why was there so little difference in the change from baseline on the LSAS? The mean differences in the LSAS, ≥30% improvement on the LSAS, and CGI-I did not diverge before 8 weeks. A longer treatment period may have shown greater separation if the dose was too low or the onset of action is slow. Faster responses within 12 weeks have been readily detected within 2–4 weeks with SSRIs or clonazepam augmentation [11, 32, 33]. If FAAH inhibition depends on structural remodeling to restore more normal functioning of the amygdala or neuronal networks [12] longer treatment may be required to demonstrate more of an effect. This suggests that inhibition of FAAH by JNJ-42165279 could influence trait anxiety more than state anxiety.

The frequency of PBO response on the CGI-I observed in this trial was 23.6% which is considered low for a trial in SAD and typically ranges from 30 to 35% [34]. Indeed, the relative consistency in PBO response rates in SAD was a reason for choosing to test for an anxiolytic effect of JNJ-42165279 in this indication. Nonetheless, the absence of a significant treatment effect on the primary endpoint for this study is unlikely to reflect excess PBO response. We had more males in the study (65%) than would be predicted based on published gender differences of SAD [35] due to the exclusion of women of childbearing potential in the first part of the study pending completion of reproductive toxicology studies.

A limitation of this study was testing a single dose of 25 mg. The 25 mg per day dose of JNJ-42165279 was selected using occupancy of the FAAH enzyme by PET and CSF measures of AEA turnover which allowed quantitation of central target engagement [5] and which were further associated with PK/PD relationships in FAAH activity and FAA turnover in blood. These data suggested that the dose selected for this study should have provided inhibition of the FAAH enzyme across the entire dosing interval. While doses up to 100 mg had been evaluated in multiple dose studies, transient elevations in liver transaminases were observed with higher doses [5]. The 25 mg dose was therefore selected as providing a favorable safety margin for proof-of-concept studies. In this study, elevations of liver transaminases were not observed in the JNJ-42165279 treatment group, less frequently than in the PBO group suggesting that the safety margin was effective although potentially too conservative.

Nonadherence to treatment occurred with 15% of the JNJ-42165279 samples having nondetectable drug concentrations during the study. Nonadherence to treatment for chronic conditions is a well-known phenomenon and can be significant [36]; nonadherence during clinical trials is common and is the focus of a variety of efforts to improve adherence [37]. A higher treatment difference in the LSAS change to baseline was observed in the sensitivity analysis censoring subjects with nondetectable drug concentrations and met the statistical threshold for effect.

Investigators were requested to ask subjects about social situations that he/she had encountered since the last visit in accordance with the instruction to challenge themselves; however, we did not collect data on adherence to the instruction. The improvement on the CGI-I provides indirect evidence of increased social engagement but quantitation was not done.

More notable was the strong correlation between plasma AEA and trough drug concentrations. While the 25 mg dose was predicted to result in substantial FAAH inhibition throughout the dosing interval, based on PK and slowly reversible covalent binding, the modeling was based on Phase 1 data with healthy subjects limited to 2 weeks of chronic dosing, which was supervised and confirmed in the Phase 1 unit. Variable adherence to dosing that may occur in an outpatient setting over a 12-week period and individual differences in absorption and metabolism could result in concentrations below that necessary for complete inhibition of the enzyme. As a clearance mechanism, a return of even a small fraction of FAAH activity could result in a substantial recovery of the metabolism of AEA. Indeed, homozygosity for the mutant A allele for rs324420 has been associated with a 50% reduction in expression of FAAH [17] and was associated in this sample with higher baseline FAA concentrations but substantially below those that can be achieved with complete inhibition. The correlation of low trough concentrations with low plasma AEA suggest that recovery of FAAH enzyme activity may occur late in the dosing interval. Complete and sustained inhibition of FAAH may be necessary for modulation of AEA to have a significant pharmacological/behavioral effect. Indeed, the FAAH inhibitor AM3506 given to mice before conditioning did not alter fear or extinction responses the next day even though enzyme activity was still 75% inhibited. They concluded that a threshold of inhibition may be required to have an effect on reductions in fear [13]. Increasing trough concentrations in order to sustain more complete inhibition of FAAH could be achieved with higher doses or through increasing the dosing frequency. Given the PK/PD results from this study, after consultation with the FDA we have elected to increase the dosing frequency to 25 mg twice daily in an ongoing proof-of-concept study in autism (https://clinicaltrials.gov/ct2/show/NCT03664232) which will substantially raise trough concentrations while modestly increasing maximal plasma concentrations.

## Conclusions

Treatment of subjects with SAD with JNJ-42165279 was associated with moderate anxiolytic effects reflected by the significantly larger percentage of subjects on active drug with greater than 30% improvement on the LSAS and improvement on the CGI-I by the end of 12 weeks of treatment. The treatment was well tolerated and was not associated with any notable clinical safety signals in this trial. The strong relationship between plasma AEA levels and trough concentrations of JNJ-42165279 suggest that escape from full FAAH inhibition occurred in subjects with lower trough concentrations allowing restoration of AEA clearance.

### Future directions

We plan to explore higher doses of JNJ-42165279 minimally raising the dose to twice daily to increase trough concentrations and the probability that complete inhibition of FAAH is sustained throughout the dosing interval. In addition to conducting a proof-of-concept study in autism we are also initiating trials in PTSD with increased doses. Adherence to behavioral instruction such as engagement in social activity could be monitored with smart phone-based apps, and we are currently evaluating the use of smart phone-based apps to monitor compliance with study drug. Given that FAAH inhibition is reported to facilitate fear extinction, this mechanism could complement cognitive behavioral treatment in contrast to the reported interference of sertraline treatment of SAD with exposure therapy [38]. We will be exploring treatment with JNJ-42165279 in combination with structured behavioral therapy in our studies in autism and PTSD.

## Funding and disclosure

This study was funded and conducted by Janssen Research & Development, LLC. The study began 15 June 2015 and was completed 9 August 2019. MES, DJP, PVDA, and LVN are full time employees of Janssen Research & Development, a division of Janssen Pharmaceutica, NV. IVH and WKS were employed by Janssen Research & Development at the time of the study and JAP, ZSS, and WCD are full time employees of Janssen Research & Development, LLC. MRL holds the copyright to the Liebowitz Social Anxiety Scale (LSAS) and Janssen Research & Development licensed it from him for use in this trial. MRL and MBS were consultants to Janssen for the trial and along with JG were investigators for the study. JG has no other disclosures relevant to the trial.

## Supplementary information

Supplementary material
