# Supplementary material for: The effects of inhibition of fatty acid amide hydrolase (FAAH) by JNJ-42165279 in social anxiety disorder: a double-blind, randomized, placebo-controlled proof-of-concept study
Source: Neuropsychopharmacology. 2020 Oct 18;46(5):1004–10. doi: 10.1038/s41386-020-00888-1 (PMC8115178; doi:10.1038/s41386-020-00888-1)
Supplement: Supplementary file 1 — Supplementary material [file 41386_2020_888_MOESM1_ESM.doc]

Figure 1S: CONSORT flow diagram

**Enrollment**

Screened for eligibility

(N =251)

Excluded (N = 102)

Screen failures N=98

Not assigned to treatment N=4

**Allocation**

**Follow up**

Placebo

N =75

JNJ-42165279

N=74

Completed 53 (70.7%)

Withdrawn 22 (29.3%)

Reasons for withdrawal

Adverse event 1 (1.3%)

Lack of efficacy 1 (1.3%)

Lost to follow-up 5 (6.7%)

Other 6 (8%)

Withdrawal by subject 9 (12%)

Completed 56 (75.7%)

Withdrawn 18 (24.3%)

Reasons for withdrawal

Adverse event 4 (5.4%)

Lack of efficacy 0

Lost to follow-up 3 (4.1%)

Other 5 (6.8%)

Withdrawal by subject 6 (8.1%)

#

The study began 15 June 2015 and completed 9 August 2018. The protocol was amended in September 2016 to add neurological examinations to confirm the safety of JNJ-42165279 following a clinical hold by the FDA on all FAAH inhibitor trials in the wake of the Bial serious adverse event (see below) and in August 2017 to allow women of child bearing potential to enroll following completion and review of segment 2 reproductive toxicology studies.

The screening period was up to 28 days, the average (s.d.) duration of screening for this study was 15.3 (7.9) days. Central randomization was implemented. At the start of the double-blind phase, subjects were randomly assigned to one of two treatment groups based on the first of two computer-generated randomization schedules prepared before the study by, or under the supervision of the sponsor. The randomization will be balanced by using randomly permuted blocks. Presence of comorbid major depressive disorder (MDD) and country will be used as stratification factors.

The investigator will not be provided with randomization codes. The codes will be maintained within the IVRS/IWRS (service provided by Bracket, now Signant Health, under contract), which has the functionality to allow the investigator to break the blind for an individual subject.

Data that may potentially unblind the treatment assignment (e.g., study medication plasma concentrations, plasma biomarkers) was handled with special care to ensure that the integrity of the blind was maintained and the potential for bias is minimized.

Under normal circumstances, the blind should not have been broken until all subjects have completed the study and the database finalized. Otherwise, the blind should be broken only if specific emergency treatment/course of action would be dictated by knowing the treatment status of the subject. In such cases, the investigator may in an emergency determine the identity of the treatment by contacting the IVRS/IWRS. It was recommended that the investigator contact the sponsor to discuss the particular situation, before breaking the blind. In the event the blind is broken, the sponsor must be informed as soon as possible. The date, time, and reason for the unblinding must be documented in the appropriate section of the case report form (CRF), and in the source document. The documentation received from the IVRS indicating the code break must be retained with the subject's source documents in a secure manner.

The blind was in fact broken by Janssen for all subjects enrolled in the trial on 16 January 2016 when news of the serious adverse event was reported from the Phase 1 SAD/MAD trial of BIA 10-2474 (manufactured by Bial) conducted by Biotrial, a contract research facility in Rennes, France. The study was unblinded and the randomization codes were provided to the investigators so that the patients could be informed about their treatment status. Safety assessments were conducted, and all safety information was analyzed, and a separate Statistical Analysis Plan was written and approved for the interim safety analysis. No efficacy data was analyzed, and datasets containing efficacy information of the subjects who were unblinded at trial suspension remained in a secure location at the data management department until final data base lock. Subjects who had their treatment assignment unblinded were continued to return for required follow up evaluations.

The 15 subjects who had to withdraw at study suspension had already been randomized and were receiving treatment at the time the study was suspended. Since results of these 15 subjects taken at the end-of-study visit were possibly biased because of the study suspension, it was decided to exclude these 15 subjects from the efficacy analysis set (the ITT set). The subjects were replaced since we wanted to meet the sample size requirements for the efficacy analysis that were specified in the protocol.

A sensitivity analysis was conducted excluding subjects from the ITT set who completed the study at trial suspension (there were 18 such subjects). Per SAP these subjects were included in the primary ITT analysis, since all their data had been collected prior to the suspension of the study. Note that the results of the 15 subjects who had to withdraw because of study suspension were not included in any of the efficacy analysis, because of the expected bias due to the reason of withdrawal.

Table 1S below shows results of all ITT subjects who entered the study after it restarted (so ITT minus the 18 who completed at study suspension).  Mean values, changes and LS mean changes are comparable to the primary efficacy results, with the difference in LS mean changes being slightly larger (-4.6) compared to the overall set (-3.8). The *P* -value reaches the protocol threshold of statistical significance at the 0.20 level (*P* =0.190). Given the similarity in the results, the overall ITT set did not appear to have been significantly impacted by the study suspension.

Table 1S

| **LSAS Total Score: Change from Baseline to Week 12 MMRM Observed Case Analysis - Sensitivity Analysis Excluding Subjects Who Completed the Study at Trial Suspension; ITT Analysis Set (Study JNJ-42165279SAX2001)** | | |
| --- | --- | --- |
|  | PLACEBO (N=59) | JNJ-42165279 25mg (N=57) |
| **Baseline** | | |
| N | 59 | 57 |
| Mean (SD) | 105.5 (16.28) | 103.2 (16.63) |
| Median (Range) | 104.0 (71,142) | 101.0 (73,140) |
| **Week 12** | | |
| N | 46 | 50 |
| Mean (SD) | 82.8 (28.79) | 72.6 (29.92) |
| Median (Range) | 84.5 (15,142) | 70.0 (10,133) |
| **Change from Baseline** | | |
| N | 46 | 50 |
| Mean (SD) | -22.1 (22.93) | -30.4 (28.22) |
| Median (Range) | -20.0 (-90,13) | -25.5 (-101,12) |
|  |  |  |
| LS Mean (SE) | -25.9 (4.09) | -30.4 (4.15) |
| P-value(minus Placebo)^a^ |  | 0.190 |
| Diff. of LS Means (SE) |  | -4.6 (5.17) |
| 60% CI |  | (-8.93,-0.18) |
| ^a^One-sided p-value with level of significance of 20%. Test for no difference between JNJ-42165279 and placebo for MMRM model with treatment (placebo, JNJ-42165279), time, time-by-treatment interaction, country and presence of comorbid MDD as factors, and baseline LSAS total score and age as a continuous covariate. Subjects who had completed the study prior to study suspension are not included in this analysis. The LSAS Total Score ranges from 0 to 144, with higher scores indicating higher probability of social anxiety disorder. | | |

Randomization codes were disclosed to the investigator if requested after the study was completed and the clinical database was closed.

Sample processing for plasma AEA: Approximately 6 ml of blood was collected in a tube with EDTA that was mixed by gently inverting the tube 8-10 times. The sample was immediately centrifuged at room temperature (minimum of 1500 x g for 15-20 minutes) until to separate cells and plasma and then placed on ice. Plasma was then distributed via pipette to equally to 4 prelabeled, pre-cooled Cryovials kept on ice. These samples were immediately frozen at -70°C until shipment to a central lab on dry ice the following day.

Assay methods for fatty acid amides: Concentration of Arachidonoyl, Oleoyl, and Palmitoyl Ethanoloamides (AEA, OEA, PEA) in human K2EDTA plasma were measured via LC/MS/MS method at Keystone Bioanalytical (North Wales, PA). The assay range is 0.1-10 ng/ml (AEA), 0.5-50 ng/ml (OEA), and 0.5-50 ng/ml (PEA). The plasma samples and isotope-labeled standards (for calibrant curve) were combined with internal standards and extracted using methyl-t-butylether. The extracted samples were dried and reconstituted with acetonitrile/water (50/50). The reconstituted samples were transferred to HPLC vial for LC/MS/MS analysis.

Genetic analysis: Genomic DNA was prepared according to standard protocol from blood samples, collected from participants. Specific DNA fragments were amplified by polymerase chain reaction and subsequently sequenced. Genotyping was carried out through the analysis of a single polymorphisms and results for only specific positions (FAAH, P129T, rs324420) by Eurofins Genomics, Ebersberg, Germany.

Drug bioanalysis: The concentration of JNJ-42165279 was quantified in plasma using a specific, validated, and sensitive liquid chromatography/tandem mass spectrometry (LC/MS/MS) method.

Adherence to treatment: There were 12 subjects identified with no detectable drug concentrations.  Ten of the 12 subjects completed the study, 2 withdrew early. One subject was lost to follow up and did not have Week 12 LSAS results so was not included in the Week 12 analysis, one subject discontinued due to an AE (GI infection, not attributed to study drug) but had LSAS measured at Day 75 and so this subject was included in the Week 12 analysis. The half-life of JNJ-42165279 at 25 mg repeat dose is 11 hours and concentrations are readily detectable for at least 48 hours after last dose (see reference 5: Postnov et al Clin Transl Sci (2018) doi:10.1111/cts.12548 for the Phase 1 data), so the absence of detectable drug suggests missing doses for several consecutive days before the visit. Compliance was determined in all subjects through pill counts and querying the subject about any differences in pills expected to be returned. Many subjects in the trial reported missing an occasional dose, there was nothing notable about the reports for those with non-detectable concentrations although determining significant non-adherence to treatment by pill counts is notoriously unreliable.

Table 2S:

Generalized Cochran-Mantel-Haenszel Test for LSAS ≥30% Improvement versus CGI Response at Week 12 for All Subjects ; ITT Analysis Set. CGI-I response is defined as a score of very much improved or improved.

|  | LSAS <30% Improvement (N=76) | | LSAS >= 30% Improvement (N=38) | |  | LSAS >= 30% Improvement versus LSAS <30% Improvement ----------- Odds Ratio ----------- | |
| --- | --- | --- | --- | --- | --- | --- | --- |
| **Parameter**  Response | n | % | n | % | Overall P-value(a) | Estimate | 95% CI |
| **CGI-I Response** |  |  |  |  |  |  |  |
| No | 69 | 90.8% | 6 | 15.8% |  |  |  |
| Yes | 7 | 9.2% | 32 | 84.2% |  |  |  |
|  | ------ |  | ------ |  |  |  |  |
| Total | 76 |  | 38 |  | <.0001 | 0.019 | (0.0059; 0.0612) |

Table 3S:

Secondary clinical endpoints change from baseline to Week 12

| Measure and Comparison | JNJ-42165279 | PBO |  | |  |
| --- | --- | --- | --- | --- | --- |
|  | Mean (SD) | Mean (SD) | Difference^1^ | 60% CI | p-value^2^ |
| HAM-A |  |  |  |  |  |
| Baseline | 10.1 (7.44) | 10.6 (7.47) |  |  |  |
| Week 12 | 6.2 (6.04) | 7.5 (7.43) |  |  |  |
| Difference | -4.2 (5.90) | -3.0 (6.20) | -0.9 | -1.75 ; -0.03 | 0.191 |
| HDRS_17_ |  |  |  |  |  |
| Baseline | 6.4 (4.95) | 6.7 (4.59) |  |  |  |
| Week 12 | 4.9 (4.83) | 5.8 (5.32) |  |  |  |
| Difference | -1.8 (3.99) | -0.9 (4.71) | -0.5 | -1.2 ; 0.15 | n.s. |
| ^1^ Difference in LS mean changes from baseline between JNJ-42165279 and PBO. | | | | | |
| ^2^ One-sided p-value with level of significance of 20%. Test for no difference between JNJ-42165279 and PBO for MMRM model with treatment (PBO, JNJ-42165279), time, time-by-treatment interaction, country and presence of comorbid MDD as factors, and baseline score and age as a continuous covariate. | | | | | |

Table 4S

LSAS Total Score Change from Baseline to Week 12 MMRM Observed Case Analysis in Subjects with Comorbid GAD; ITT Analysis Set

|  | | |
| --- | --- | --- |
|  | PLACEBO (N=10) | JNJ-42165279 25mg (N=10) |
| **Baseline** | | |
| N | 10 | 10 |
| Mean (SD) | 104.7 (12.07) | 110.9 (16.78) |
| Median (Range) | 105.0 (82,119) | 110.0 (88,137) |
| **Week 12** | | |
| N | 10 | 9 |
| Mean (SD) | 86.4 (18.70) | 70.4 (24.09) |
| Median (Range) | 88.5 (47,108) | 72.0 (20,93) |
| **Change from Baseline** | | |
| N | 10 | 9 |
| Mean (SD) | -18.3 (10.00) | -39.9 (32.74) |
| Median (Range) | -16.5 (-40,-4) | -34.0 (-89,5) |
|  |  |  |
| LS Mean (SE) | -18.4 (6.96) | -35.0 (7.26) |
| P-value(minus Placebo)^a^ |  | 0.059 |
| Diff. of LS Means (SE) |  | -16.6 (10.09) |
| 60% CI |  | (-25.30,-7.90) |
| ^a^One-sided *P* -value with level of significance of 20%. Test for no difference between JNJ-42165279 and placebo for MMRM model with treatment (placebo, JNJ-42165279), time, time-by-treatment interaction, country and presence of comorbid MDD as factors, and baseline LSAS total score and age as a continuous covariate. The LSAS Total Score ranges from 0 to 144, with higher scores indicating higher probability of social anxiety disorder. | | |

Table 5S:

Exploratory clinical endpoints

| Measure and Comparison | JNJ-42165279 | PBO |  |  |  |
| --- | --- | --- | --- | --- | --- |
|  |  |  | Difference^1^ | 60% CI | *P*-value^2^ |
| GAD-7 |  |  |  |  |  |
| Baseline | 10.3 (6.33) | 10.5 (5.34) |  |  |  |
| Week 12 | 5.9 (5.25) | 7.9 (5.38) |  |  |  |
| Difference | -4.5 (4.78) | -2.2 (5.55) | -1.9 (0.82) | (-2.6, -1.22) | 0.01 |
| SDS |  |  |  |  |  |
| Baseline | 16.7 (7.00) | 17.2 (6.91) |  |  |  |
| Week 12 | 11.7 (7.48) | 11.9 (8.30) |  |  |  |
| Difference | -5.3 (6.38) | -4.9 (7.04) | -0.8 (1.27) | (-1.87, 0.27) | 0.26 |
| SHAPS |  |  |  |  |  |
| Baseline | 3.3 (3.68) | 3.6 (3.27) |  |  |  |
| Week 12 | 1.6 (2.52) | 2.2 (2.67) |  |  |  |
| Difference | -1.7 (2.89) | -1.1 (2.88) | -0.6 (0.39) | (-0.93,-0.27) | 0.06 |
| MOS-Sleep R (9 item) |  |  |  |  |  |
| Baseline | 45.1 (11.73) | 44.5 (10.44) |  |  |  |
| Week 12 | 51.4 (9.22) | 48.7 (9.78) |  |  |  |
| Difference | 6.6 (7.80) | 4.1 (7.39) | 2.5 (1.19) | (1.51, 3.52) | 0.02 |
| QLES |  |  |  |  |  |
| Baseline | 51.6 (18.35) | 52.8 (16.33) |  |  |  |
| Week 12 | 62.7 (18.09) | 60.9 (17.87) |  |  |  |
| Difference | 10.8 (12.55) | 7.7 (15.32) | 2.3 (2.43) | (0.27, 4.37) | 0.17 |
| SATE |  |  | Estimate^3^ |  |  |
| condition improved | 56.3% | 48.4% | 0.73 |  | 0.38 |
| mood improved | 46.9% | 37.1% | 1.50 |  | 0.27 |
| activities improved | 51.6% | 45.2% | 1.29 |  | 0.47 |
| sleep improved | 32.8% | 24.2% | 1.53 |  | 0.29 |
| ^1^ Difference in LS mean changes from baseline between JNJ-42165279 and PBO. | | | | | |
| ^2^ One-sided *P* -value with level of significance of 20%. Test for no difference between JNJ-42165279 and PBO for MMRM model with treatment (PBO, JNJ-42165279), time, time-by-treatment interaction, country and presence of comorbid MDD as factors, and baseline score and age as a continuous covariate.  ^3^ Generalized Cochran-Mantel-Haenszel Test for row mean scores differ | | | | | |

GAD-7=Generalized Anxiety Disorder 7; SDS=Sheehan Disability Scale; SHAPS=Snaith Hamilton Pleasure Scale scored by item (range 0-14); MOS-Sleep R=Medical Outcome Scale for Sleep Revised; subscales were transformed to T-values, higher values indicate better sleep (fewer reported problems); QLES= Quality of Life Enjoyment and Satisfaction; higher scores indicate greater QoL; SATE= Self-Assessment of Treatment Experience.

Figure 2S. Plasma AEA, OEA, and PEA by treatment group: A,B, C respectively.


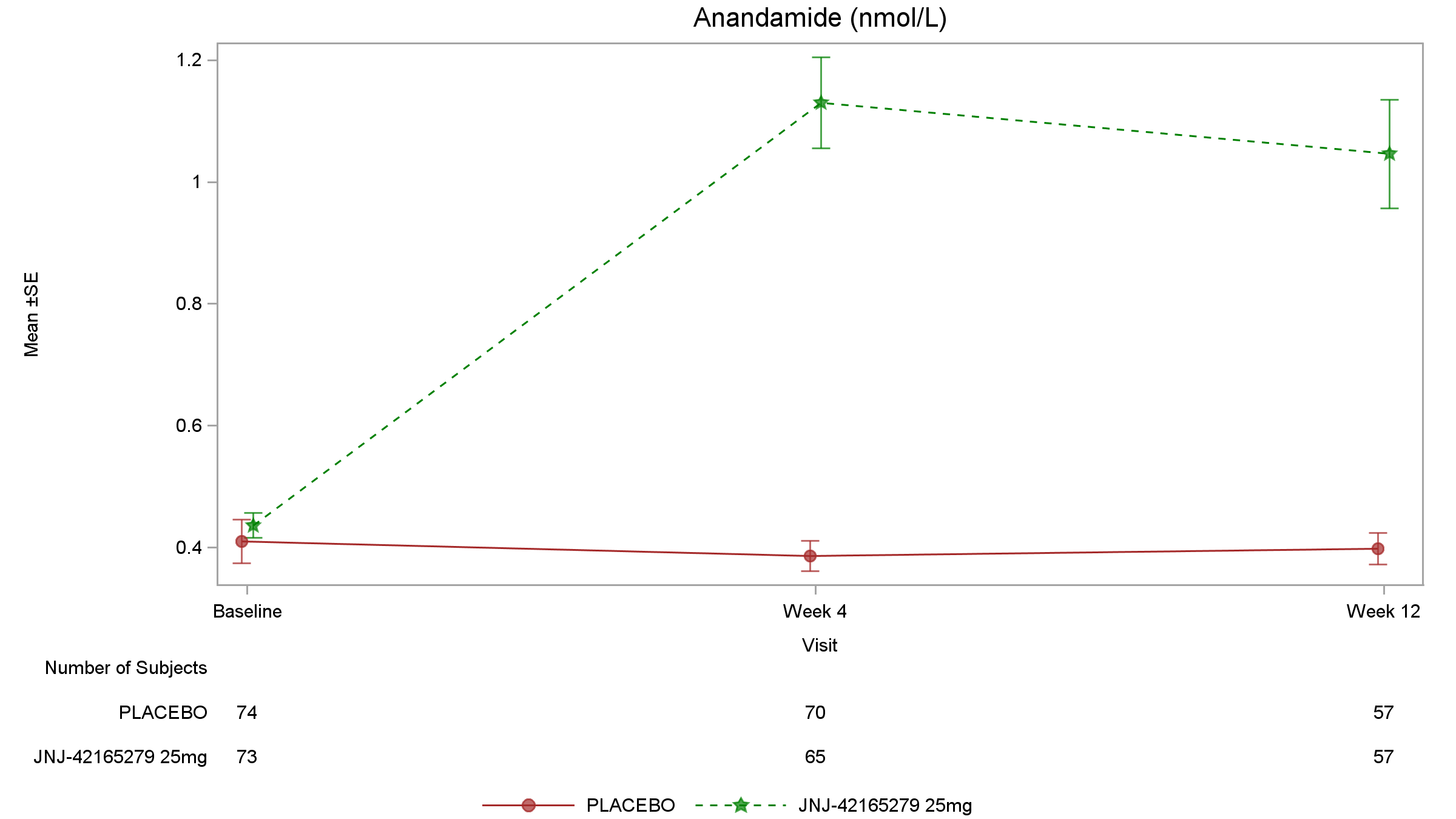


A


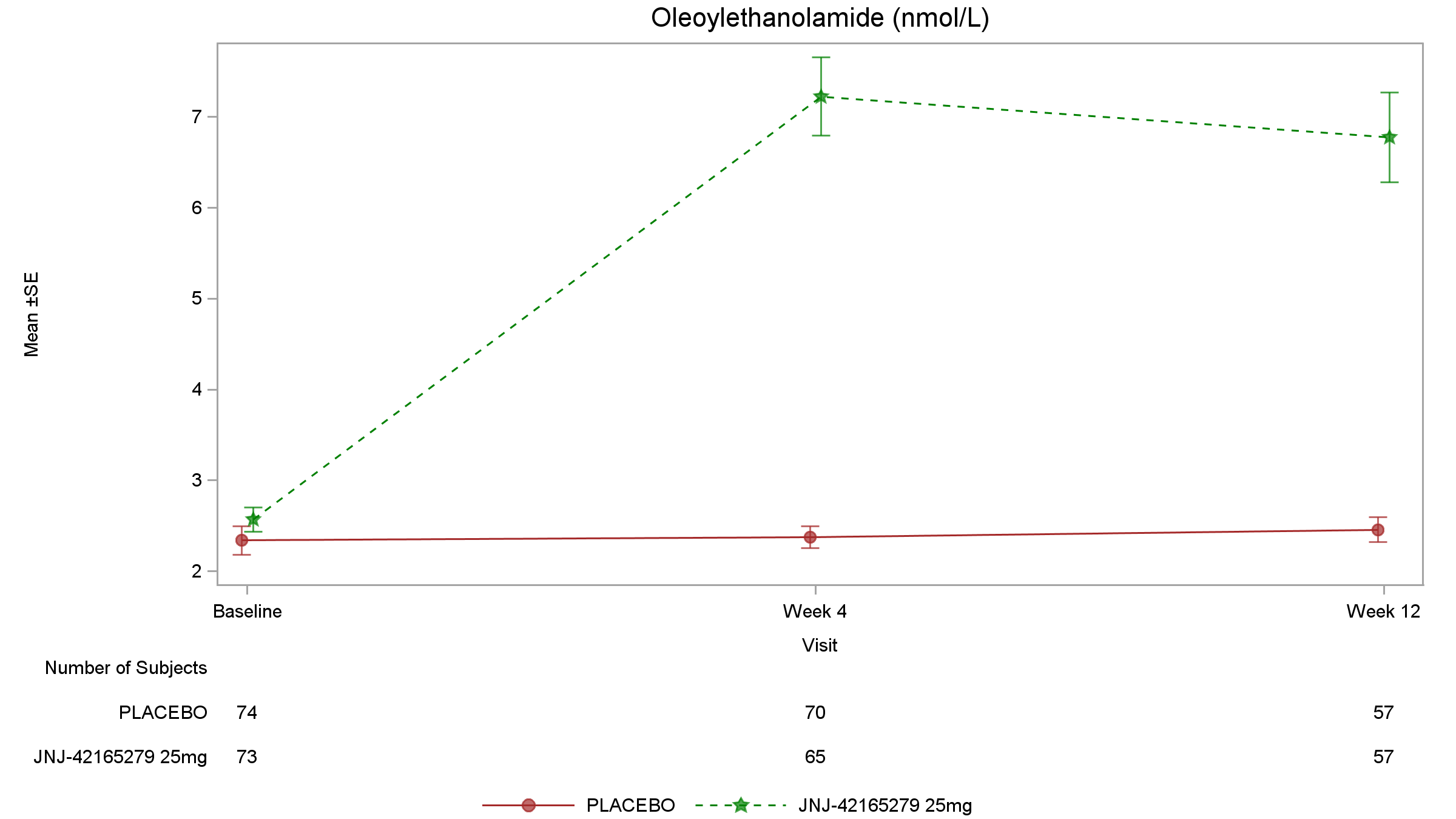


B


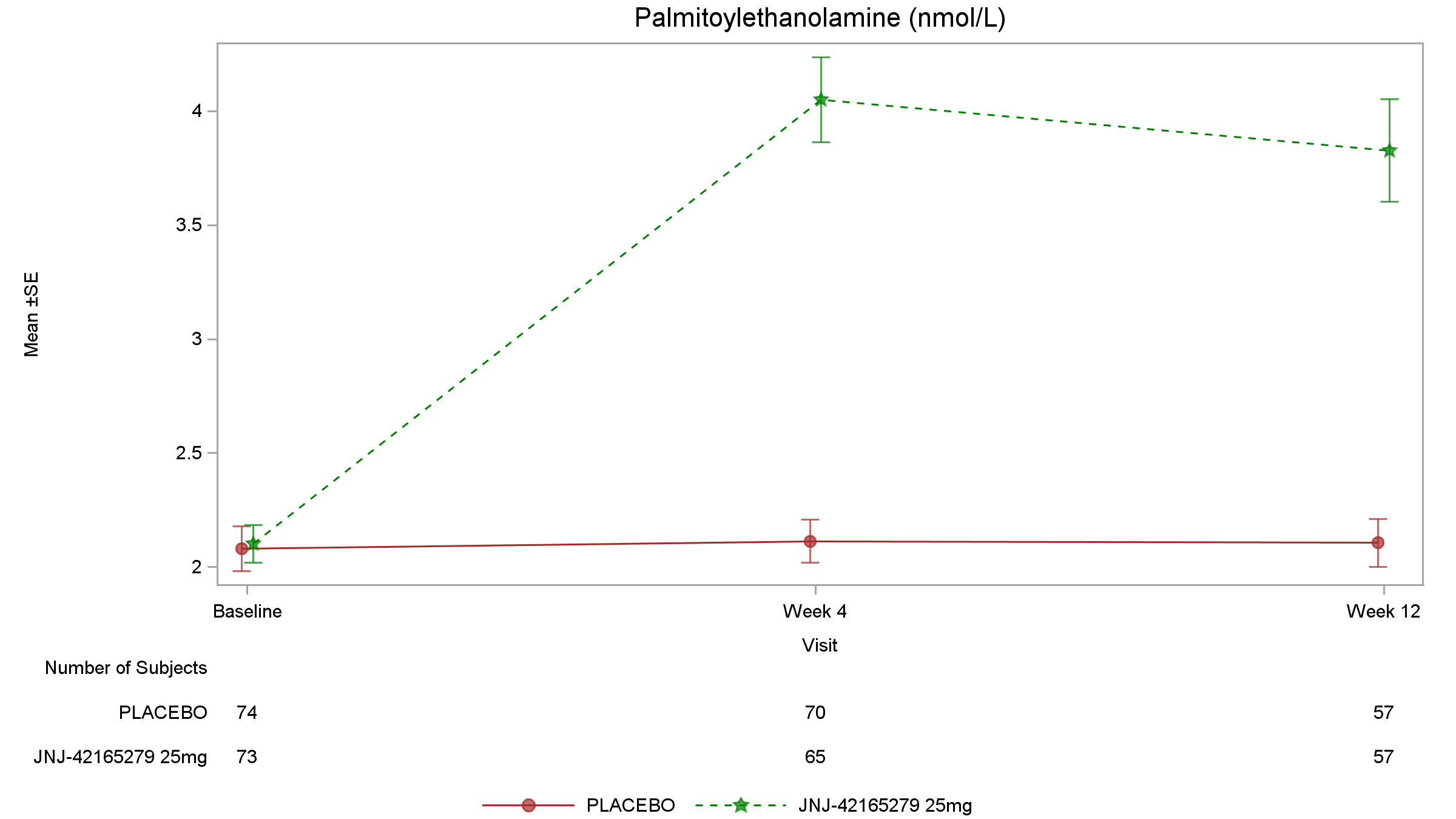


C

Figure 3S: Week 12 LSAS change from baseline JNJ42165279 subjects grouped into tertiles of Week 12 plasma AEA concentrations vs. PBO.

First tertile

Second tertile

Third tertile

PBO

Tertiles are for the JNJ-42165279 treatment arm only. Treated subjects with most elevated AEA (3^rd^ tertile) at Week 12 exhibited the numerically greatest change from baseline with a 12.9 point greater change compared to PBO, although this difference was not statistically significant. The nominal *P*-value for a two sided t-test was *P*=0.17 (uncorrected for multiple comparisons). Similar results were observed when using a median split, rather than tertiles. Analysis excludes subjects with drug concentration below 1 ng/ml at Day 14 or at Day 28.

Table 6S: Baseline Plasma FAA By Genotype for All Subjects (PBO and JNJ-42165279 Combined); Safety Analysis Set

| Parameter Observed Genotype | N | Mean | SE | SD | Median | Min | Max | P-value versus A/A Wilcoxon rank sum test |
| --- | --- | --- | --- | --- | --- | --- | --- | --- |
| **Anandamide (nmol/L)** |  |  |  |  |  |  |  |  |
| **All Subjects (N=141)** |  |  |  |  |  |  |  |  |
| A/A | 11 | 2.05 | 0.564 | 1.870 | 1.80 | 0.6 | 7.5 |  |
| A/C | 41 | 1.13 | 0.073 | 0.470 | 1.01 | 0.3 | 2.8 | 0.0131 |
| C/C | 89 | 1.14 | 0.050 | 0.469 | 1.07 | 0.4 | 3.3 | 0.0106 |
|  |  |  |  |  |  |  |  |  |
| **Oleoylethanolamide (nmol/L)** |  |  |  |  |  |  |  |  |
| **All Subjects (N=141)** |  |  |  |  |  |  |  |  |
| A/A | 11 | 13.46 | 2.311 | 7.664 | 11.65 | 6.1 | 34.5 |  |
| A/C | 41 | 7.16 | 0.496 | 3.177 | 6.39 | 2.7 | 15.9 | 0.0002 |
| C/C | 89 | 6.93 | 0.284 | 2.679 | 6.45 | 2.3 | 16.5 | <0.0001 |
|  |  |  |  |  |  |  |  |  |
| **Palmitoylethanolamine (nmol/L)** |  |  |  |  |  |  |  |  |
| **All Subjects (N=141)** |  |  |  |  |  |  |  |  |
| A/A | 11 | 10.15 | 1.107 | 3.672 | 9.11 | 5.8 | 19.4 |  |
| A/C | 41 | 6.41 | 0.268 | 1.715 | 6.18 | 3.2 | 11.4 | <0.0001 |
| C/C | 89 | 6.84 | 0.272 | 2.570 | 6.16 | 2.2 | 16.1 | 0.0004 |
|  |  |  |  |  |  |  |  |  |

Samples were tested for the rs324420 genotype in 141 subjects in the safety set; samples from 6 subjects were not available due to missed collections, missing or declined informed consent, or handling error. The A/A genotype was present in 11 subjects (8%); A/C in 41 (29%); and C/C in 89 (63%). Baseline FAAs (AEA, PEA, OEA) were higher in the A/A group compared to the other 2 groups. The treatment patterns did not significantly differ between the 3 groups.

Table 7S: Treatment-Emergent Adverse Events by Body System or Organ Class and Dictionary-Derived Term; Safety Analysis Set

|  | PBO (N=75) | JNJ-42165279 25mg (N=74) |
| --- | --- | --- |
| **Body System**  Preferred Term | n ( % ) | n ( % ) |
|  | | |
| **Total no. Subjects with Adverse Events** | 42 ( 56.0) | 46 ( 62.2) |
|  | | |
| **Nervous System Disorders** | 17 ( 22.7) | 19 ( 25.7) |
| Headache | 13 ( 17.3) | 15 ( 20.3) |
| Dizziness | 0 | 3 ( 4.1) |
| Dizziness Postural | 0 | 1 ( 1.4) |
| Dysaesthesia | 1 ( 1.3) | 1 ( 1.4) |
| Hyporeflexia | 0 | 1 ( 1.4) |
| Cervical Radiculopathy | 1 ( 1.3) | 0 |
| Cognitive Disorder | 2 ( 2.7) | 0 |
| Migraine | 1 ( 1.3) | 0 |
| Paraesthesia | 1 ( 1.3) | 0 |
|  | | |
| **Infections And Infestations** | 12 ( 16.0) | 14 ( 18.9) |
| Upper Respiratory Tract Infection | 3 ( 4.0) | 5 ( 6.8) |
| Nasopharyngitis | 6 ( 8.0) | 4 ( 5.4) |
| Gastroenteritis | 0 | 1 ( 1.4) |
| Gastroenteritis Norovirus | 0 | 1 ( 1.4) |
| Pharyngitis Streptococcal | 0 | 1 ( 1.4) |
| Sinusitis | 1 ( 1.3) | 1 ( 1.4) |
| Urinary Tract Infection | 0 | 1 ( 1.4) |
| Bacterial Vaginosis | 1 ( 1.3) | 0 |
| Hordeolum | 1 ( 1.3) | 0 |
| Influenza | 1 ( 1.3) | 0 |
| Vulvovaginal Candidiasis | 1 ( 1.3) | 0 |
|  | | |
| **Gastrointestinal Disorders** | 11 ( 14.7) | 10 ( 13.5) |
| Diarrhoea | 2 ( 2.7) | 5 ( 6.8) |
| Constipation | 2 ( 2.7) | 2 ( 2.7) |
| Dry Mouth | 3 ( 4.0) | 2 ( 2.7) |
| Abdominal Discomfort | 0 | 1 ( 1.4) |
| Abdominal Pain Upper | 0 | 1 ( 1.4) |
| Flatulence | 0 | 1 ( 1.4) |
| Proctalgia | 0 | 1 ( 1.4) |
| Dental Caries | 1 ( 1.3) | 0 |
| Food Poisoning | 2 ( 2.7) | 0 |
| Gingival Pain | 1 ( 1.3) | 0 |
| Nausea | 4 ( 5.3) | 0 |
| Vomiting | 1 ( 1.3) | 0 |
|  | | |
| **General Disorders And Administration Site Conditions** | 4 ( 5.3) | 8 ( 10.8) |
| Fatigue | 3 ( 4.0) | 6 ( 8.1) |
| Chest Pain | 1 ( 1.3) | 1 ( 1.4) |
| Influenza Like Illness | 0 | 1 ( 1.4) |
|  | | |
| **Psychiatric Disorders** | 9 ( 12.0) | 8 ( 10.8) |
| Libido Decreased | 0 | 2 ( 2.7) |
| Aggression | 0 | 1 ( 1.4) |
| Alcohol Use Disorder | 0 | 1 ( 1.4) |
| Anxiety | 1 ( 1.3) | 1 ( 1.4) |
| Bruxism | 0 | 1 ( 1.4) |
| Depression | 0 | 1 ( 1.4) |
| Initial Insomnia | 0 | 1 ( 1.4) |
| Insomnia | 6 ( 8.0) | 1 ( 1.4) |
| Irritability | 0 | 1 ( 1.4) |
| Depressed Mood | 2 ( 2.7) | 0 |
| Middle Insomnia | 1 ( 1.3) | 0 |
| Sleep Disorder | 1 ( 1.3) | 0 |
|  | | |
| **Investigations** | 6 ( 8.0) | 5 ( 6.8) |
| Blood Glucose Increased | 1 ( 1.3) | 1 ( 1.4) |
| Blood Pressure Increased | 0 | 1 ( 1.4) |
| Haematocrit Decreased | 0 | 1 ( 1.4) |
| Heart Rate Increased | 0 | 1 ( 1.4) |
| Liver Function Test Increased | 1 ( 1.3) | 1 ( 1.4) |
| Lymphocyte Morphology Abnormal | 0 | 1 ( 1.4) |
| Platelet Count Increased | 0 | 1 ( 1.4) |
| Alanine Aminotransferase Increased | 2 ( 2.7) | 0 |
| Blood Creatine Increased | 1 ( 1.3) | 0 |
| Blood Triglycerides Increased | 1 ( 1.3) | 0 |
| Blood Uric Acid Increased | 1 ( 1.3) | 0 |
| Weight Increased | 1 ( 1.3) | 0 |
|  | | |
| **Renal And Urinary Disorders** | 1 ( 1.3) | 3 ( 4.1) |
| Haematuria | 0 | 3 ( 4.1) |
| Pollakiuria | 1 ( 1.3) | 0 |
|  | | |
| **Respiratory, Thoracic And Mediastinal Disorders** | 3 ( 4.0) | 3 ( 4.1) |
| Epistaxis | 0 | 1 ( 1.4) |
| Nasal Congestion | 2 ( 2.7) | 1 ( 1.4) |
| Rhinorrhoea | 0 | 1 ( 1.4) |
| Dyspnoea | 1 ( 1.3) | 0 |
| Oropharyngeal Pain | 1 ( 1.3) | 0 |
|  | | |
| **Immune System Disorders** | 2 ( 2.7) | 2 ( 2.7) |
| Anaphylactic Reaction | 0 | 1 ( 1.4) |
| Seasonal Allergy | 0 | 1 ( 1.4) |
| Allergy to Arthropod Bite | 1 ( 1.3) | 0 |
| Food Allergy | 1 ( 1.3) | 0 |
|  | | |
| **Injury, Poisoning And Procedural Complications** | 3 ( 4.0) | 2 ( 2.7) |
| Laceration | 0 | 1 ( 1.4) |
| Spinal Compression Fracture | 0 | 1 ( 1.4) |
| Vaccination Complication | 0 | 1 ( 1.4) |
| Ligament Sprain | 1 ( 1.3) | 0 |
| Muscle Strain | 1 ( 1.3) | 0 |
| Nail Injury | 1 ( 1.3) | 0 |
| Thermal Burn | 1 ( 1.3) | 0 |
|  | | |
| **Musculoskeletal And Connective Tissue Disorders** | 5 ( 6.7) | 2 ( 2.7) |
| Muscle Spasms | 0 | 1 ( 1.4) |
| Myalgia | 0 | 1 ( 1.4) |
| Arthralgia | 2 ( 2.7) | 0 |
| Arthritis | 1 ( 1.3) | 0 |
| Muscular Weakness | 1 ( 1.3) | 0 |
| Musculoskeletal Pain | 2 ( 2.7) | 0 |
| Neck Pain | 1 ( 1.3) | 0 |
|  | | |
| **Metabolism And Nutrition Disorders** | 3 ( 4.0) | 1 ( 1.4) |
| Fluid Retention | 0 | 1 ( 1.4) |
| Decreased Appetite | 1 ( 1.3) | 0 |
| Hypoglycaemia | 1 ( 1.3) | 0 |
| Lactose Intolerance | 1 ( 1.3) | 0 |
|  | | |
| **Skin And Subcutaneous Tissue Disorders** | 3 ( 4.0) | 1 ( 1.4) |
| Eczema | 0 | 1 ( 1.4) |
| Acne | 2 ( 2.7) | 0 |
| Skin Discolouration | 1 ( 1.3) | 0 |
|  | | |
| **Vascular Disorders** | 1 ( 1.3) | 1 ( 1.4) |
| Hypertension | 1 ( 1.3) | 1 ( 1.4) |
|  | | |
| **Blood And Lymphatic System Disorders** | 2 ( 2.7) | 0 |
| Leukopenia | 1 ( 1.3) | 0 |
| Neutropenia | 2 ( 2.7) | 0 |
|  | | |
| **Ear And Labyrinth Disorders** | 2 ( 2.7) | 0 |
| Vertigo | 2 ( 2.7) | 0 |
|  | | |
| **Eye Disorders** | 3 ( 4.0) | 0 |
| Dry Eye | 2 ( 2.7) | 0 |
| Vision Blurred | 1 ( 1.3) | 0 |
|  | | |
| **Neoplasms Benign, Malignant And Unspecified (Incl Cysts And Polyps)** | 1 ( 1.3) | 0 |
| Colon Adenoma | 1 ( 1.3) | 0 |
|  | | |
| **Reproductive System And Breast Disorders** | 2 ( 2.7) | 0 |
| Breast Mass | 1 ( 1.3) | 0 |
| Ejaculation Delayed | 1 ( 1.3) | 0 |
|  | | |
| **Surgical And Medical Procedures** | 1 ( 1.3) | 0 |
| Sinus Operation | 1 ( 1.3) | 0 |
| Note: Percentages calculated with the number of subjects in each group as denominator. Note: Reported dictionary version: MedDRA 19.1 | | |
